# Response of Purslane Plants Grown under Salinity Stress and Biostimulant Formulations

**DOI:** 10.3390/plants13172431

**Published:** 2024-08-30

**Authors:** Mostafa H. M. Mohamed, Maha Mohamed Elsayed Ali, Reda M. Y. Zewail, Vasiliki Liava, Spyridon A. Petropoulos

**Affiliations:** 1 Department of Horticulture, Faculty of Agriculture, Benha University, Moshtohor, Toukh 13736, Egypt; mustafa.muhammed@fagr.bu.edu.eg; 2 Department of Soil and Water Sciences, Faculty of Agriculture, Benha University, Moshtohor, Toukh 13736, Egypt; maha.aly@fagr.bu.edu.eg; 3 Botany Department, Faculty of Agriculture, Benha University, Benha 13736, Egypt; reda.zewail@fagr.bu.edu.eg; 4 Laboratory of Vegetable Production, University of Thessaly, Fytokou Street, 38446 Volos, Greece; vasiliki.liava@gmail.com

**Keywords:** putrescine, salicylic acid, wild edible species, proline, nitrate, carotenoids, chlorophylls

## Abstract

Purslane has been suggested as an alternative crop suitable for human consumption due to its high content of minerals, omega-3 fatty acids, and several health-beneficial compounds. In this study, we aimed to evaluate the effect of salinity stress (tap water (control), 2000, 4000, 6000, 8000, and 10,000 mg L^−1^), biostimulant application (putrescine and salicylic acid at 200 mg L^−1^), and the combination of the tested factors (i.e., salinity × biostimulant application) on the growth and chemical composition of purslane plants (*Portulaca oleracea* L.) over two growing seasons (2022 and 2023). Irrigation with tap water and putrescine application resulted in the highest plant height, weight of aboveground and underground parts, and number of shoots per plant. In contrast, the lowest values of growing parameters were recorded under severe saline stress (10,000 mg L^−1^), especially for the plants that were not treated with biostimulants. The same trends were observed for macronutrients (N, P, K), total carbohydrates, total chlorophylls, and vitamin C content in leaves. Moreover, nitrate and proline content was higher in plants grown under salinity stress, especially under severe stress (8000–10,000 mg L^−1^) without biostimulant application. In general, the application of biostimulants mitigated the negative impact of salinity on plant growth and leaf chemical composition, while the effect of putrescine on the tested parameters was more beneficial than that of salicylic acid. In conclusion, this study provides useful information regarding the use of putrescine and salicylic acid as biostimulatory agents with the aim of increasing purslane growth under salinity conditions.

## 1. Introduction

Salinity and drought are among the factors most limiting crop production in many regions of the world. The main factors responsible for salinity are the use of irrigation water of low quality, the excessive fertilization of crops, and deficient drainage of soils [1]. Salinity is a significant abiotic stressor, particularly in arid and semi-arid regions throughout the Mediterranean basin, where access to irrigation water of high quality is restricted [2]. The increasing demand for water in the agricultural sector requires the use of water of marginal quality, for instance desalinated seawater or brackish water, which may contain salt residues [3]. However, the continuous application of saline water for irrigation purposes can lead to the buildup of salts near the soil surface [4], thus affecting crops due to osmotic and ionic stress, which disrupt cellular functions [5,6], as well as due to gradual soil salinization [7]. In particular, osmotic stress may influence the ability of plant cells to detoxify reactive oxygen species (ROS), which are synthesized under stress conditions [8]. Similarly, high salinity in soils may disorder the ability of roots to absorb water, thus inhibiting plant physiological and biochemical processes [9]. Therefore, salinity stress reduces plant photosynthetic rates, affects primary and secondary metabolites [5], and causes nutritional and hormonal imbalances [10] due to increased intracellular osmotic pressure and to ion toxicity from sodium (Na^+^) and chloride (Cl^−^) accumulation in plant tissues [2].

Recently, biostimulants, including various classes of compounds with variable mechanisms of action, have been used as an ecofriendly agronomic tool to support the growth of plants under abiotic stressors such as salinity [11,12,13]. In this context, the ubiquitous organic poly-cations known as polyamines (PAs) are associated with several essential roles in various cellular, biochemical, and metabolic processes; they are crucial under stress conditions and for the senescence response of plants [14]. Putrescine is the main product of the PA biosynthetic pathway and serves as a precursor for the synthesis of spermidine and spermine [15]. Exogenous application of PAs, such as putrescine, may result in improved tolerance to abiotic stress such as drought and salinity and could be used as an effective tool to mitigate stress and to increase crop yield and quality without any severe effects on crops or the environment [16]. Putrescine may also reduce Na^+^ buildup, boost antioxidant activities, and improve the photosynthetic capacity of plants subjected to salinity stress while protecting the plasma membrane by scavenging ROS [16].

On the other hand, salicylic acid is an organic compound and an endogenous regulator of plant hormones that can affect the physiological and biochemical processes of plants [17]. It can also regulate many plant processes at the gene level and improve plant tolerance to biotic and abiotic stress [18]. It may also enhance various processes like seed germination, photosynthesis, and the biosynthesis of chlorophyll and other pigments, as well as increase enzymatic and non-enzymatic antioxidant activity and the activity of ROS scavenger enzymes [19]. Moreover, salicylic acid increases plant development through stomatal regulation and ion uptake and transport [20]. Therefore, several studies suggest that salicylic acid is an endogenous signaling molecule that may trigger plant defense and their responses to pathogen attacks locally and through a systemic acquired resistance (hypersensitive response) [21]. According to the studies of Li et al. [21] and Arfan et al. [22], spraying salicylic acid on *Torreya grandis* and wheat plants was able to mitigate the growth inhibition caused by moderate salinity stress. Under saline conditions, this compound has healing and growth-promoting effects by protecting plants against oxidative damage and impaired photosynthetic efficiency induced by stress conditions [23].

*Portulaca oleracea* L., commonly known as purslane, is a wide-spread weed belonging to the Portulacaceae family [24]. Recently, it has been suggested for the production of edible leafy greens due to its high nutritional value [25]. Its leaves and stems contain phenolic compounds, vitamins, essential amino acids, and proteins, and are also rich in minerals, and omega-3 fatty acids [26,27,28]. Omega-3 fatty acids may protect against cancer, heart diseases, and several other chronic illnesses and ailments that affect modern people [29]. The lack of vegetable sources rich in omega-3 fatty acids has resulted in suggesting this species as an alternative cultivated vegetable that can grow even in harsh conditions with minimum agronomic inputs and fulfill the nutrient requirements in human diets [30,31]. Therefore, the present study aimed to assess the effect of salinity stress and putrescine and salicylic acid application on purslane plant growth and leaf chemical composition, as well as to evaluate the potential of using the tested compounds for the mitigation of the adverse effects of salinity on plants.

## 2. Results and Discussion

### 2.1. Vegetative Growth Parameters

All the vegetative growth parameters were recorded for two consecutive years (Table 1). Salinity level and biostimulant application significantly affected the tested traits, while there was a significant interaction between the tested factors. Regarding salinity stress, there was a negative effect on vegetative growth traits as the salinity level increased. In particular, plant height, number of shoots, and fresh and dry weights of plants were decreased up to the highest tested salinity level (10,000 mg L^−1^) at which the lowest values of growth parameters were recorded. This trend was confirmed in both growing seasons. Moreover, the control treatment (plants treated with tap water) did not differ significantly from the lowest salinity level (2000 mg L^−1^) for all the studied traits and for both years, except for plant height in 2022. Previously, Bekmirzaev et al. [32] and Hassanpouraghdam et al. [33] suggested that salinity may negatively affect purslane plant height and weight, since the decrease in the osmotic potential of plant cells resulted in a significant decrease in water uptake and further led to a significant reduction in fresh weight in stressed plants [34].

Regarding biostimulant application, putrescine and salicylic acid had a positive impact on all the tested vegetative growth parameters and for both growing years, without significant differences between the two biostimulants (except for the number of shoots/plant in 2022 where no differences were recorded between all the treatments (including the control treatment). In particular, the highest plant height (26.3 and 30.0 cm), number of shoots (11.9 and 13.6), fresh weight (261 and 280 g), and dry weight (38.4 and 46.1 g) were recorded for putrescine treatment (in 2022 and 2023 growing year, respectively), followed by salicylic acid and the control treatment. Polyamines such as putrescine have been associated with increased plant growth and development since they act as plant growth regulators [35], plant hormones [36], or they may activate signaling molecules that regulate stomatal closure [37]. Salicylic acid is an organic substance that occurs naturally in plants and is endogenously generated as a signaling molecule that affects a variety of physiological and biochemical processes in plants, and it can serve as an important signaling molecule of defense mechanisms against biotic and abiotic stressors [38]. It also plays a vital role in nitrogen assimilation and the plant–water relationship [18], thus improving tolerance to salt and drought stress [38]. Moreover, its exogenous application has been associated with an improved shelf-life of vegetable products due to delayed ripening and reduced chlorophyll degradation [36].

Regarding the combinatorial effect of the tested factors, a significant interaction was recorded (Table 1). Specifically, in both years, the maximum values of height (35.6 and 37.1 cm), shoot number/plant (15.8 and 17.3), herb fresh weight (317 and 341 g), and herb dry weight (47.2 and 56.5 g) were observed in plants irrigated with tap water and sprayed with putrescine, without statistically significant differences from the respective plants treated with salicylic acid. On the contrary, the lowest values were recorded in plants grown under the highest salinity treatment (10,000 mg L^−1^) without biostimulant application, followed by those sprayed with salicylic acid at the same salinity level. In general, the negative effects of salinity stress on the vegetative growth parameters of purslane plants were mitigated by putrescine application, with increasing trends being recorded compared to the other treatments. Moreover, the fresh weight of putrescine-treated plants was significantly higher than untreated plants for all the salinity treatments, thus indicating the stress-mitigating effects of this particular biostimulant on purslane plants. According to the literature, the application of putrescine and salicylic acid mitigated the negative effects of salinity on vegetative growth in various plant species such as Korean ginseng (*Panax ginseng* Meyer) [39], foxtail millet (*Setaria italica* L.) [40], and cucumber seedlings (*Cucumis sativus* L.) [41]. Salicylic acid also alleviated the harmful effects of high salinity by inducing the biosynthesis of growth hormones like auxins and cytokinins, which further decreased the uptake of toxic ions [18]. Moreover, this compound can boost the antioxidant defense system and ionic homeostasis, thus improving plant growth by minimizing the deleterious effects of salinity stress [42]. In general, polyamines may trigger cellular metabolism and modulate plant physiology and facilitate the mitigation of the negative effect of salinity since they can influence plant development processes, including cell division, embryogenesis, the development of flowers and reproductive organs, fruit ripening, root growth, and leaf senescence [43,44]. They also serve as signaling molecules in the abscisic acid-regulated stress response pathway and they can interact with macromolecules like RNA, DNA, translation and transcription complexes, and cell membranes to help plants tolerate and overcome stress conditions [45]. According to Shu et al. [46], putrescine application may alleviate the disruption of photosynthetic apparatus due to high salinity through the regulation of proteins of thylakoid membrane expression and protection against lipid peroxidation.

### 2.2. Root Growth Parameters

Data in Table 2 present the effects of salinity and biostimulant application on root growth parameters. Our results indicate that increasing salinity levels negatively affected the fresh and dry weight of purslane roots, which were significantly decreased by increasing salinity. Therefore, the highest fresh and dry weight of roots were recorded in plants irrigated with tap water, while the lowest values of these traits were observed in plants irrigated with the highest salinity level, since increasing salinity at levels higher than 2000 mg L^−1^ significantly reduced the fresh and dry weight of roots. Similarly to our study, Hassanpouraghdam et al. [33] also reported the negative effect of salinity on the growth of purslane roots. According to the literature, salinity stress is one of the most harmful abiotic stressors which affects the growth and productivity of crops due to osmotic stress and ion toxicity, leading to secondary oxidative stress [18]. Moreover, the foliar application of putrescine had a beneficial effect on root growth parameters, which were significantly higher than those of plants treated with both salicylic acid and the control treatment, while salicylic acid application had a less notable effect, although also being significantly higher than the control treatment (Table 2). Polyamines such as putrescine may directly regulate cell division during root formation and increase root biomass, while indirectly, they may regulate root growth through interaction with ethylene and auxins [47].

A significant interaction between the tested factors regarding their effect on root growth parameters was also recorded. In most cases, the foliar application of putrescine or salicylic acid resulted in significantly increased fresh and dry weight of purslane roots compared to the untreated plants, while the highest values were recorded for plants treated with putrescine and irrigated with tap water or at 2000 mg L^−1^ (only for root fresh weight in 2022). In general, putrescine application resulted in higher values of root growth parameters compared to both salicylic acid and tap water; however, in most cases, they differed significantly only from the tap water treatment and no significant differences were recorded between the biostimulant formulations. Therefore, our results indicate that the application of putrescine and salicylic acid may alleviate the adverse effect of salinity on root growth, especially the application of putrescine, which resulted in higher values in most cases. Similarly to our study, salinity stress significantly affected the fresh and dry weight of roots in ginseng plants, while spraying putrescine resulted in similar values to those of non-stressed plants, indicating the stress-mitigating properties of putrescine [39]. According to Rathinapriya et al. [40] and Buffagni et al. [43], putrescine may influence plant growth recovery after stress through the modulation of root architecture and increased root density, which facilitate tolerance to high salinity. Moreover, the foliar spraying of salicylic acid may increase the biomass of roots by altering biochemical and physiological processes [48], and it seems to have both healing and growth-promoting impacts on roots when plants are grown under saline conditions [23].

### 2.3. Chemical Composition

In both growing seasons, there was a negative effect of increasing salinity on macronutrient and carbohydrate content (Table 3), whereas no significant differences were recorded between the control treatment (tap water) and salinity levels up to 4000 mg L^−1^ in most cases. Salinity stress can severely affect plants through the production of reactive oxygen species (ROS) such as hydrogen peroxide (H_2_O_2_) and superoxide (O_2_) [49]. Moreover, the uptake of nutrients can be inhibited under salinity stress due to competitive interactions between Na^+^ and other ions [18]. Hence, as the level of salinity stress increased, the content of macronutrients was reduced until it reached the lowest content at the highest salinity level (10,000 mg L^−1^). A similar trend was recorded for carbohydrate content, which was significantly reduced at moderate to severe stress levels (6000 to 10,000 mg L^−1^). According to the literature, a varied response of carbohydrate content has been reported depending on the species and the severity of stress [50]; mild salinity stress has been recorded as an eliciting strategy to improve the quality of specific vegetable products through the accumulation of carbohydrates which are related to the taste of the edible product [51]. In contrast to our study, Teixeira and Carvalho [52] reported a significant increase in total carbohydrate content in purslane leaves under severe stress (up to 24.2 dS m^−1^), and similar results were recorded by Uddin et al. [53], who subjected purslane plants to EC levels of up to 264 mM of NaCl. These contradicting results between our study and the literature reports could be due to either genotypic differences in the purslane plants tested among the different studies or to differences in the stage of plants when they were subjected to stress and the stress duration. For example, in the study of Teixeira and Carvalho [52], plants were irrigated for 1 or 2 weeks with saline water at 6 weeks after transplantation, whereas Uddin et al. [53] applied salinity stress for 10 and 20 days at 8 weeks after sowing. In our study, a prolonged stress was implemented (40 d), which justifies the different response of plants compared to the abovementioned studies, since according to the literature, prolonged stress and high salinity intensity may have severe effects even on salt-tolerant species whose mechanisms cannot compensate for the negative long-term impact of stressors [53].

Data presented in Table 3 also indicate that N, P, K, and the total carbohydrate content of leaves were significantly increased by spraying plants with the tested biostimulants, particularly for putrescine application, which resulted in higher content than salicylic acid and the control treatment in almost every case (except for N content, where no significant differences between putrescine and salicylic acid were detected). Statistical analysis also showed a significant interaction between the tested factors for the abovementioned parameters. In both years, purslane plants treated with putrescine and irrigated with tap water had the highest values of N, P, K, and total carbohydrates, followed by the plants irrigated with saline water (up to 4000 mg L^−1^), where putrescine application also resulted in higher macronutrient and carbohydrate contents than in the salicylic acid and the control treatments in most cases. According to the literature, salicylic acid may enhance the uptake of essential elements that are necessary for plant growth [54] and also increase carbohydrate production by increasing chlorophyll content and plant photosynthetic activity [55]. Carbohydrate content can also be increased by putrescine application [39], while polyamines such as putrescine may improve the root structure, thus leading to increased nutrient absorption and nutrient content in plant tissues [47].

Table 4 presents the results for the effect of the tested factors on total chlorophyll, vitamin C, nitrate, and proline content in purslane leaves. Regarding the total chlorophyll and vitamin C content, low to moderate levels of salinity (2000 to 4000 mg L^−1^ and 2000 to 6000 mg L^−1^ for 2022 and 2023, respectively) resulted in increased values of the studied parameters, whereas the lowest values were recorded for the highest salinity level (10,000 mg L^−1^). This effect could be due to the protective role of ascorbic acid in plants, which increases with increasing salinity up to the point where this particular defense mechanism is disrupted and plants cannot overcome the stress conditions [18]. Ma et al. [56] suggested that extreme salinity stress may reduce photosynthesis and result in growth suppression in addition to poor photosynthetic apparatus development and ROS formation. Moreover, high levels of salts in leaves increase the activity of chlorophyll oxidase enzymes such as chlorophyllase, which restrict chlorophyll synthesis [18,57] and reduce the content of photosynthetic pigments and the overall plant vigor [40,42,48]. In contrast, the opposite trend was observed for nitrate and proline content, which increased with increasing salinity, especially at moderate to high salinity levels (Table 4). Proline is a water-soluble amino acid that plays an important role in regulating redox through osmotic adjustment [39], which explains its increased contents under salinity stress, since the production of osmolytes is an inherent defense mechanism of plants when they try to cope with salinity stress through maintaining high water content in plant tissues [40,42,58]. Moreover, proline has also been reported to suppress ROS formation, decrease the detrimental effect of salinity on thylakoid membranes, and increase nitrogen fixation in plants [48].

Regarding the effect of the tested biostimulants, the greatest chlorophyll content was recorded after putrescine application, followed by salicylic acid, whereas the content of vitamin C and nitrate did not differ among the treatments. Finally, proline content was reduced after the application of putrescine and salicylic acid; thus, indicating the stress mitigating effects of the tested biostimulants. The interaction of biostimulant application and salinity stress showed that putrescine protected chlorophyll more efficiently than salicylic acid, especially at moderate to high salinity levels (Table 4). On the other hand, vitamin C was not affected by biostimulant treatments, regardless of salinity level, except for the case of high salinity, where the lowest values were recorded regardless of the biostimulant treatment. Nitrate content was significantly increased at salinity levels higher than 6000 mg L^−1^, with no significant differences among the biostimulant treatments, whereas putrescine and salicylic acid resulted in the lowest nitrate content when plants were irrigated with tap water. Similarly, the highest proline content was recorded at high salinity levels (8000 and 10,000 mg L^−1^) for non-treated plants (no biostimulants added).

According to the literature, the application of polyamines led to a significantly higher content of photosynthetic pigments in tomato seedlings compared to untreated control plants grown under salinity stress [59]. This might be due to the antioxidant role of putrescine, as it stabilizes macromolecules and cell membranes and thus plays a protective role against chlorophyll degradation under stress conditions [55]. The same trend was observed for salicylic acid, which did not differ significantly from putrescine and untreated plants at low to moderate salinity (up to 6000 mg L^−1^), whereas at high salinity (>8000 mg L^−1^), it did not differ from untreated plants. In previous studies, salicylic acid enhanced photosynthetic pigment content in berseem plants (*Trifolium alexandrinum*) by suppressing the formation of ROS, mainly H_2_O_2_ accumulation [42]. This compound may also control the stomatal opening, lower the transpiration rate under salinity stress, raise the efficiency of the antioxidant machinery, and decrease the formation of ROS [60]. ROS regulation is associated with an increased expression of genes related to antioxidant enzymes which activate the antioxidant defense system and induce plant tolerance to salinity stress by maintaining higher water content in leaves [54]. Moreover, it might prevent chlorophyll degradation or promote the activity of enzymes related to chlorophyll biosynthesis [56,58]. Haghshenas et al. [55] also reported that putrescine and salicylic acid application (alone or combined) may increase vitamin C content compared to untreated plants grown under saline conditions, although lower contents of vitamin C should be expected at high salinity levels. Finally, the application of salicylic acid and polyamines may reduce proline accumulation under salinity stress since it serves as an osmoprotectant and increases under stress conditions to preserve cell turgor pressure and inhibit cell damage [40,60,61].

## 3. Materials and Methods

### 3.1. Experimental Conditions

This study was conducted over two successive growing seasons (2022 and 2023) at the experimental farm (30°16′17.2 ″N 30°46′20.4 ″E) of the College of Agriculture, Benha University, Qalubia Governorate, Egypt. Pot experiments were conducted aiming to assess the effect of salinity stress and biostimulant application on purslane plant growth. During the growing seasons, the average values of temperature, precipitation, and relative humidity ranged between 16 and 38 °C, 1.8 and 5.4 mm, and 55 and 69%, respectively. The soil pH was 7.72 and the texture was clay loam. The remaining soil physicochemical parameters are presented in Table 5. The physical parameters were determined according to Jackson [62], while the chemical analyses were carried out according to the protocols of Black et al. [63]. Soil pH and EC were determined in the soil paste, whereas organic matter content was determined using potassium chromate and then titrated by ferrous sulfate [63].

### 3.2. Plant Material and Experimental Layout

In both growing seasons, a local variety of purslane (*Portulaca oleracea* L.; Egyptian purslane, cv. Balady) was used and the seeds were sown on 1 April in 4 L plastic pots filled with clay loam soil (see Table 5). Fifteen days after sowing, the seedlings were thinned, leaving one seedling per pot. The plants were allowed to recover from thinning shock for 10 days and then they were irrigated with tap water at regular intervals. At 25 days after sowing, the salinity experiment was initiated consisting of the following six salinity levels: control (tap water), 2000, 4000, 6000, 8000, and 10,000 mg L^−1^. The desired levels of salinity were achieved with the addition of NaCl (Merck, Darmstadt, Germany) in tap water. In each pot, 200 mL of tap or saline water were applied every two days, until the end of the study. The first dose of the tested biostimulants was applied foliarly until run-off at 30 days after sowing and four foliar sprays were conducted at intervals of 7 days until the end of the experiment. The experimental design was laid out according to a complete randomized block design (CRBD) with 18 treatments representing the combination of two factors. The first factor was saline irrigation with six levels and the second factor was biostimulant application with three treatments. All the treatments were replicated three times and each replicate consisted of twenty pots.

#### 3.2.1. Salinity Treatments

The implemented salinity treatments were as follows:

1. Control: irrigated with tap water; 250 mg L^−1^ (4.28 mM NaCl);
2. Salinity level 2000 mg L^−1^: irrigated with tap water containing 2000 mg L^−1^ NaCl (34.2 mM NaCl);
3. Salinity level 4000 mg L^−1^: irrigated with tap water containing 4000 mg L^−1^ NaCl (68.4 mM NaCl);
4. Salinity level 6000 mg L^−1^: irrigated with tap water containing 6000 mg L^−1^ NaCl (102.7 mM NaCl);
5. Salinity level 8000 mg L^−1^: irrigated with tap water containing 8000 mg L^−1^ NaCl (136.9 mM NaCl);
6. Salinity level 10,000 mg L^−1^: irrigated with tap water containing 10,000 mg L^−1^ NaCl (171.2 mM NaCl).

#### 3.2.2. Biostimulant Foliar Spraying

The experimental treatments of biostimulants were as follows:

1. Control: spraying with tap water;
2. Putrescine spraying at a concentration of 200 mg L^−1^;
3. Salicylic acid spraying at a concentration of 200 mg L^−1^.

The selected concentrations of putrescine and salicylic acid were selected based on preliminary experiments that were performed by our team aiming to identify the best treatments to be implemented in the present work.

### 3.3. Data Recorded

#### 3.3.1. Vegetative Growth Measurements

Plant growth parameters such as plant height (using a measurement tape), shoot number, and fresh and dry aboveground and root weight were measured (using a precision balance; KERN 440-35N; KERN & SOHN GmbH; Balingen, Germany) in each pot. Whole plants were harvested at 65 days after sowing, washed with tap water, and kept in a cool, dry place, and then fresh weight was recorded. After that, samples were transferred into an oven and left until dry, and dry weight was recorded with a precision balance (KERN 440-35N; KERN & SOHN GmbH; Balingen, Germany).

#### 3.3.2. Chemical Composition

For both growing seasons, the chemical composition of leaves was determined after 65 days of sowing. On the day of harvest, plants were collected and transferred to the lab for chemical analyses. The total chlorophyll content of leaves was measured according to the protocol described by the Association of Official Analytical Chemists [64]. Chlorophyll was extracted using 20 mL of 80% acetone until the residue became colorless, and then the absorption was recorded at 663 and 645 nm using a spectrophotometer (Model UV752/UV754-single beam UV/Vis spectrophotometer, YK Scientific, Shanghai, China). Total N, P, and K of the samples were determined after digestion with a mixture of strong acids (HClO_4_ and H_2_SO_4_ at a ratio of 1:3 (*v*:*v*)) using the Microkjeldahl digestion and flame photometer apparatus (JENWAY, PEP-7 Jenway, Dunmow, UK) as described by Pregl [65], John [43], and Brown and Lilleland [66], respectively. Total carbohydrate content was measured in the dry samples according to Herbert et al. [67]. Briefly, 0.2 g of dry sample was extracted with HCl (0.1 M) in Eppendorf tubes (Eppendorf North America; Enfield, CT, USA) (25 mL) for 4 h. Then, the extract was filtrated and placed into a flask. After that, 1 mL of the extract was mixed with 1 mL of concentrated sulphuric acid and 1 mL of phenol 5% and chlorophyll content was measured at 490 nm using a spectrophotometer at 490 nm (Model UV752/UV754-single beam UV/Vis spectrophotometer, YK Scientific, Shanghai, China). The ascorbic acid content of leaves was determined by titration using the indicator 2,6 dichlorophenol indophenol as described by the Association of Official Analytical Chemists [64]. Finally, leaf proline content was determined according to the Association of Official Analytical Chemists [64].

### 3.4. Statistical Analysis

The experiment was carried out according to a completely randomized design (CRD) with three replications per treatment. Prior to analysis, data were checked for following normal distribution using the Shapiro–Wilk test and then analyzed with a two-way analysis of variance (ANOVA) using the criterion of least significant difference (LSD) for mean comparisons (*p* = 0.05). The statistical software used was JMP v. 16.1 (SAS Institute Inc., Cary, NC, USA).

## 4. Conclusions

The results of the present study highlighted that purslane plants can be grown under salinity stress conditions, although severe salinity negatively affects plant growth and the chemical composition of leaves. On the other hand, putrescine and salicylic acid application at a rate of 200 mg L^−1^ promoted plant development under non-stress conditions, indicating that these compounds have biostimulatory properties and could be used to increase the biomass yield of purslane plants. Under salinity stress conditions (up to 10,000 mg L^−1^ NaCl), foliar spraying with putrescine and salicylic acid alleviated the adverse effects of salinity on plant growth. Therefore, it could be suggested that the application of putrescine and salicylic acid is a sustainable strategy to cultivate purslane as an alternative/complementary crop under saline conditions, under which most of the conventional vegetable crops cannot survive.

## Figures and Tables

**Table 1:** Effect of salinity level and exogenous application of biostimulants on vegetative growth traits of purslane plants during 2022 and 2023 seasons.

Treatments		Height (cm)		Shoots/Plant (No)		Fresh Weight/Plant (g)		Dry Weight/Plant (g)	
Salinity Level	Biostimulants	2022	2023	2022	2023	2022	2023	2022	2023
Tap water		31.8	34.0	13.9	15.2	284	308	42.2	50.7
2000 mg L^−1^		28.1	33.6	13.4	15.3	279	304	41.4	50.3
4000 mg L^−1^		26.2	30.2	12.1	12.7	255	285	37.9	46.8
6000 mg L^−1^		22.5	26.8	10.8	11.5	225	240	33.4	39.1
8000 mg L^−1^		20.2	22.8	8.9	10.0	194	210	30.0	34.6
10,000 mg L^−1^		17.3	17.2	7.5	8.5	163	176	23.9	28.4
LSD at 0.05		2.14	1.92	1.12	1.09	14.3	12.9	3.15	3.18
	Control	22.5	24.3	10.4	10.9	198	216	29.3	35.3
	Putrescine	26.3	30.0	11.9	13.6	261	280	38.4	46.1
	Salicylic acid	24.2	28.0	11.1	12.1	241	265	36.3	43.5
	LSD at 0.05	3.06	2.75	1.60	1.56	20.45	18.45	4.50	4.55
Tap water	Control	28.3	29.4	12.3	13.1	245	259	36.4	42.3
	Putrescine	35.6	37.1	15.8	17.2	317	341	47.2	56.5
	Salicylic acid	31.4	35.6	13.7	15.3	289	324	43.1	53.2
2000 mg L^−1^	Control	26.9	28.7	12.1	13.4	239	251	35.2	41.4
	Putrescine	29.4	36.9	14.9	17.0	308	335	46.0	55.6
	Salicylic acid	28.1	35.1	13.2	15.6	291	326	43.1	53.8
4000 mg L^−1^	Control	24.3	27.1	11.9	12.5	216	236	32.1	38.9
	Putrescine	27.8	32.4	12.4	13.6	284	315	42.2	51.8
	Salicylic acid	26.4	31.0	12.1	12.1	264	304	39.4	49.7
6000 mg L^−1^	Control	21.1	24.3	10.2	10.6	182	204	27.0	33.1
	Putrescine	24.1	29.4	11.3	12.5	251	269	37.2	44.0
	Salicylic acid	22.3	26.7	10.8	11.3	242	246	36.1	40.2
8000 mg L^−1^	Control	19.2	21.6	8.6	8.4	167	189	24.9	31.0
	Putrescine	21.8	24.8	9.2	11.6	218	224	33.1	36.9
	Salicylic acid	19.6	21.9	9.0	9.9	198	217	32.0	35.8
10,000 mg L^−1^	Control	15.3	14.8	7.1	7.3	138	156	20.2	25.1
	Putrescine	18.9	19.3	7.8	9.8	186	198	27.4	32.0
	Salicylic acid	17.6	17.4	7.6	8.4	164	174	24.1	28.2
LSD at 0.05		5.3	4.8	2.8	2.7	35	32	7.8	7.9

Comparison of means for each growing season was performed using the least significant difference (LSD) test at *p* = 0.05. Means in the same column and for the same parameter (i.e., salinity, biostimulants application, and their combination) with a difference higher than the respective LSD value are significantly different.

**Table 2:** Effect of salinity level and exogenous application of biostimulants on root growth parameters of purslane plants during 2022 and 2023 seasons.

Treatments		Roots Fresh Weight/Plant (g)		Roots Dry Weight/Plant (g)	
Salinity Level	Biostimulants	2022	2023	2022	2023
Tap water		52.8	59.3	8.69	10.43
2000 mg L^−1^		51.6	58.2	8.52	10.24
4000 mg L^−1^		48.7	52.3	8.15	9.21
6000 mg L^−1^		41.8	45.5	6.85	8.16
8000 mg L^−1^		35.4	37.0	5.91	6.39
10,000 mg L^−1^		26.7	30.2	4.33	5.15
LSD at 0.05		2.3	3.1	0.28	0.43
	Control	38.4	40.7	6.37	7.09
	Putrescine	47.2	52.1	7.80	9.18
	Salicylic acid	42.9	49.1	7.06	8.52
	LSD at 0.05	3.3	3.3	0.40	0.61
Tap water	Control	38.4	51.8	8.14	9.14
	Putrescine	47.2	64.7	9.46	11.46
	Salicylic acid	42.9	61.3	8.48	10.68
2000 mg L^−1^	Control	46.3	50.2	7.61	8.92
	Putrescine	56.9	62.6	9.24	11.12
	Salicylic acid	51.6	61.9	8.71	10.67
4000 mg L^−1^	Control	42.6	45.3	7.12	8.14
	Putrescine	54.2	58.2	9.16	10.24
	Salicylic acid	49.3	53.4	8.16	9.26
6000 mg L^−1^	Control	36.6	39.8	6.08	6.92
	Putrescine	47.3	51.3	7.69	9.11
	Salicylic acid	41.6	48.5	6.79	8.46
8000 mg L^−1^	Control	31.2	30.6	5.24	5.12
	Putrescine	38.2	41.5	6.41	7.13
	Salicylic acid	36.7	39.0	6.09	6.92
10,000 mg L^−1^	Control	24.8	26.4	4.02	4.28
	Putrescine	29.3	34.1	4.81	6.02
	Salicylic acid	25.9	30.2	4.15	5.14
LSD at 0.05		5.7	5.7	0.69	1.06

Comparison of means for each growing season was performed using the least significant difference (LSD) test at *p* = 0.05. Means in the same column and for the same parameter (i.e., salinity, biostimulants application, and their combination) with a difference higher than the respective LSD value are significantly different.

**Table 3:** Effect of salinity level and exogenous application of biostimulants on macronutrient and carbohydrate content of purslane leaves during 2022 and 2023 seasons.

Treatments		N%		P%		K%		Total Carbohydrates%	
Salinity Level	Biostimulants	2022	2023	2022	2023	2022	2023	2022	2023
Tap water		1.58	1.55	0.237	0.246	1.42	1.54	17.1	18.7
2000 mg L^−1^		1.57	1.52	0.235	0.243	1.42	1.51	16.4	18.1
4000 mg L^−1^		1.52	1.45	0.236	0.240	1.37	1.47	16.1	17.1
6000 mg L^−1^		1.41	1.35	0.219	0.216	1.28	1.32	14.6	14.6
8000 mg L^−1^		1.29	1.22	0.191	0.196	1.16	1.13	13.1	12.3
10,000 mg L^−1^		1.18	1.72	0.184	0.180	1.09	1.05	11.2	11.3
LSD at 0.05		0.07	0.06	0.007	0.008	0.05	0.06	1.1	1.2
	Control	1.33	1.27	0.203	0.201	1.15	1.19	12.7	13.1
	Putrescine	1.52	1.47	0.231	0.238	1.41	1.47	16.9	17.8
	Salicylic acid	1.43	1.38	0.217	0.221	1.30	1.35	14.6	15.0
	LSD at 0.05	0.10	0.09	0.010	0.011	0.07	0.09	1.6	1.7
Tap water	Control	1.46	1.39	0.219	0.227	1.24	1.31	14.6	15.8
	Putrescine	1.72	1.70	0.256	0.264	1.62	1.74	19.8	21.7
	Salicylic acid	1.58	1.56	0.238	0.249	1.41	1.58	16.7	18.6
2000 mg L^−1^	Control	1.43	1.41	0.214	0.225	1.26	1.28	14.2	15.3
	Putrescine	1.69	1.65	0.252	0.261	1.59	1.71	19.3	21.0
	Salicylic acid	1.61	1.52	0.241	0.243	1.43	1.54	15.9	17.9
4000 mg L^−1^	Control	1.41	1.32	0.226	0.219	1.21	1.29	13.7	14.6
	Putrescine	1.62	1.62	0.246	0.256	1.52	1.68	18.4	20.1
	Salicylic acid	1.54	1.41	0.236	0.246	1.38	1.46	16.2	16.8
6000 mg L^−1^	Control	1.36	1.24	0.204	0.196	1.14	1.17	12.4	11.9
	Putrescine	1.46	1.43	0.236	0.241	1.39	1.42	16.8	17.6
	Salicylic acid	1.41	1.38	0.219	0.212	1.32	1.38	14.7	14.3
8000 mg L^−1^	Control	1.21	1.17	0.182	0.176	1.08	1.09	11.2	10.8
	Putrescine	1.39	1.28	0.201	0.211	1.24	1.18	14.9	14.2
	Salicylic acid	1.27	1.21	0.190	0.202	1.17	1.14	13.1	11.9
10,000 mg L^−1^	Control	1.12	1.09	0.173	0.167	1.02	1.01	10.2	10.4
	Putrescine	1.26	1.17	0.197	0.195	1.14	1.10	12.4	12.7
	Salicylic acid	1.17	1.14	0.182	0.178	1.11	1.04	11.0	10.8
LSD at 0.05		0.17	0.16	0.017	0.019	0.12	0.15	2.8	3.0

Comparison of means for each growing season was performed using the least significant difference (LSD) test at *p* = 0.05. Means in the same column and for the same parameter (i.e., salinity, biostimulants application, and their combination) with a difference higher than the respective LSD value are significantly different.

**Table 4:** Effect of salinity level and exogenous application of biostimulants on chemical composition of purslane leaves during 2022 and 2023 seasons.

Treatments		Total Chlorophylls (mg/100 g FW)		Vitamin C Content (mg/100 g FW)		Nitrate Content (mg/g FW)		Proline Content (μg/g FW)	
Salinity Level	Biostimulants	2022	2023	2022	2023	2022	2023	2022	2023
Tap water		166	170	69.0	74.1	2.28	2.21	467	445
2000 mg L^−1^		172	174	70.7	76.8	2.37	2.27	649	522
4000 mg L^−1^		180	181	77.2	80.6	2.79	2.61	793	646
6000 mg L^−1^		168	174	80.3	83.3	3.28	3.14	1035	888
8000 mg L^−1^		154	156	71.9	75.7	3.39	3.33	1392	1342
10,000 mg L^−1^		144	144	63.5	62.9	3.11	3.05	1511	1433
LSD at 0.05		8	7	5.3	5.0	0.24	0.21	73	92
	Control	143	149	71.2	73.5	3.00	2.90	1096	991
	Putrescine	188	187	73.3	78.1	2.75	2.69	922	802
	Salicylic acid	162	164	71.9	75.2	2.86	2.71	906	845
	LSD at 0.05	12	10	7.5	7.1	0.34	0.30	104	132
Tap water	Control	149	154	68.4	71.5	2.41	2.34	483	452
	Putrescine	181	185	69.7	76.8	2.19	2.11	458	436
	Salicylic acid	169	171	68.9	74.2	2.26	2.19	462	448
2000 mg L^−1^	Control	153	159	69.3	74.8	2.52	2.46	694	673
	Putrescine	192	189	72.4	79.8	2.24	2.17	612	441
	Salicylic acid	173	174	70.6	76.0	2.37	2.18	642	453
4000 mg L^−1^	Control	162	164	76.2	76.9	2.92	2.83	865	792
	Putrescine	198	197	78.3	84.3	2.68	2.58	732	564
	Salicylic acid	182	183	77.2	80.7	2.78	2.43	784	584
6000 mg L^−1^	Control	141	152	79.4	81.3	3.41	3.21	1108	984
	Putrescine	201	204	81.3	86.4	3.14	3.07	984	817
	Salicylic acid	162	168	80.2	82.4	3.29	3.14	1014	864
8000 mg L^−1^	Control	132	139	71.2	75.2	3.62	3.49	1640	1462
	Putrescine	184	182	72.9	76.9	3.19	3.21	1320	1246
	Salicylic acid	148	149	71.8	75.2	3.38	3.30	1216	1319
10,000 mg L^−1^	Control	124	128	62.4	61.8	3.14	3.09	1790	1586
	Putrescine	172	164	65.2	64.2	3.08	3.02	1426	1311
	Salicylic acid	137	141	63.1	62.8	3.12	3.06	1318	1402
LSD at 0.05		21	17	13.1	12.3	0.59	0.52	180	228

Comparison of means for each growing period was performed with the least significant difference (LSD) test at *p* = 0.05. Means in the same column and for the same parameter (i.e., salinity, biostimulants application, and their combination) with a difference higher than the respective LSD value are significantly different.

**Table 5:** Physical and chemical parameters of the soil used in the two growing seasons.

Physical Parameters		Chemical Parameters			
		Cations		Anions	
Coarse sand	7.13%	Ca^2+^	6.26 mEq/L	CO_3_^−^	0.00 mEq/L
Fine sand	17.27%	Mg^2+^	3.02 mEq/L	HCO_3_^−^	3.92 mEq/L
Silt	22.28%	Na^+^	5.36 mEq/L	Cl^−^	4.42 mEq/L
Clay	53.32%	K^+^	0.93 mEq/L	SO4^−^	7.38 mEq/L
Soil pH	7.72	Available N		26.4 mg/kg	
E.C.	1.48 dS/m	Available P		8.73 mg/kg	
Organic matter	1.59%	Available K		119.9 mg/kg	

## Data Availability

Data are available upon request.
